# The complete chloroplast genome of *Agropyron pectinatum* (M. Bieb.) P. Beauv.

**DOI:** 10.1080/23802359.2021.1959451

**Published:** 2021-07-28

**Authors:** Yijing Luo, Junfeng Yang, Wenxuan Du, Yongzhen Pang

**Affiliations:** a Institute of Animal Sciences, Chinese Academy of Agricultural Sciences, Beijing, China; b College of Grassland Sciences, Qingdao Agricultural University, Qingdao, China

**Keywords:** *Agropyron pectinatum*, Chloroplast genome

## Abstract

*Agropyron pectinatum* is a perennial forage widely cultivated in China, and it belongs to the Gramineous family. In this study, we assembled the complete chloroplast genome of *A. pectinatum*. The whole chloroplast genome of *A. pectinatum* is 135,041 bp in length, comprising a pair of inverted repeat (IR) regions (20,821 bp) that are separated by a large single copy (LSC) region (80,632 bp) and a small single copy (SSC) region (12,767 bp). The chloroplast genome of *A. pectinatum* contains 133 genes, and 87 of them are protein-coding genes, 38 are tRNA, and eight are rRNA genes. The chloroplast genome of *A. pectinatum* could provide valuable information for varieties identification and evolution of the *Agropyron* Gaertn.

*Agropyron pectinatum* (M. Bieb.) P. Beauv. (Palisot de Beauvois, 1812) is a warm-loving and light-loving xerophyte, and it belongs to the *Agropyrong* Gaertn. Plants of the *Agropyrong* Gaertn are perennial forage of the Gramineous family (Alevtina and Aydar 2014; Li et al. 2020), and they are high-quality forage with high yield and high feeding value. Besides, *Agropyrong* also possesses strong adaptability and resistance with very important ecological value, and they can grow well in regions with an annual rainfall of 230–380 mm (Song 1992). The chloroplast is an important organelle of plants, and it has been a focus of research in plant molecular evolution and systematics for decades (Clegg et al. 1994). However, up to now, the chloroplast genome sequences of *A. pectinatum* are still unavailable. In the present study, we sequenced and assembled the chloroplast genome of *A. pectinatum*, and further characterized its gene structure features. Taken together, the complete chloroplast genome sequence of *A. pectinatum* will be a valuable resource for further investigation on species taxonomy and molecular evolution within the *Agropyron* Gaertn.

The *A. pectinatum* plant used in this study were grown and the corresponding voucher specimen (No. 16530) was deposited at the Herbarium of the Institute of Animal Sciences of the Chinese Academy of Agricultural Sciences, Beijing, China (E116°29′, N40°03′, http://ias.caas.cn/, Yongzhen Pang, pangyongzhen@caas.cn). The total genomic DNA of *A. pectinatum* was extracted from the young leaves by using the modified CTAB method (Doyle and Doyle 1987) and used for the shotgun library construction. After cluster generation, library was sequenced on an Illumina Hiseq 2000 platform and 150 bp paired-end reads were generated. The software GetOrganelle v1.5 (Jin et al. 2018) was used to assemble the cleaned reads into a complete chloroplast genome, with the chloroplast genome of *Avena sativa* (GenBank accession number: NC_027468) as a reference. The chloroplast genome annotation was performed through the online program CPGAVAS2 (Shi et al. 2019) and GeSeq (Tillich et al. 2017), followed by manual correction. The assembled chloroplast genome sequence has been submitted to the GenBank database under the accession number MW309815.

It was found that the complete chloroplast genome of *A. pectinatum* is 135,041 bp in length and it contains two inverted repeat (IRa and IRb) regions of 20,821 bp, which was separated by a large single-copy (LSC) region of 80,632 bp and a small single-copy (SSC) region of 12,767 bp. The total GC content of the complete chloroplast genome, LSC, SSC, IR regions are 38.34%, 59.71%, 9.45%, and 15.42% respectively. The complete chloroplast genome contains 133 genes, including 87 protein-coding genes, 38 tRNA, and eight rRNA genes. Among them, six are ATP synthase related genes (*atpI*, *atpH*, *atpF*, *atpA*, *atpE*, and *atpB*), one is involved in carbon fixation pathway (*rbcL*), four genes are involved in nucleotide metabolism (*rpoB*, *rpoC1*, *rpoC2*, and *rpoA*), 16 genes encoding electron transport (*ndhB*, *ndhH*, *ndhA*, *ndhI*, *ndhG*, *ndhD*, *ndhF*, *ndhB*, *petN*, *ndhJ*, *ndhK*, *ndhC*, *petA*, *petG*, *petB*, and *petD*), five genes encoding light collection structural protein (PSI) (*psaC*, *psaB*, *psaA*, *psaI*, and *psaJ*), 14 genes encoding light collection structural protein (PSII) (*psbA*, *psbK*, *psbI*, *psbD*, *psbC*, *psbZ*, *psbM*, *psbJ*, *psbL*, *psbF*, *psbE*, *psbB*, *psbT* and *psbH*), four genes encoding photosynthesis-related protein (*ndhE*, *ccsA*, *petL,* and *pbf1*), two genes encoding possible PSI structural protein (*ycf3* and *ycf4*), 36 genes encoding amino acid transfer protein (*trnH-GUG*, *trnM-CAU*, *trnL-CAA*, *trnV-GAC*, *trnA-UGC*, *trnR-ACG*, *trnN-GUU*, *trnL-UAG*, *trnN-GUU*, *trnR-ACG*, *trnA-UGC*, *trnV-GAC*, *trnL-CAA*, *trnM-CAU*, *trnH-GUG*, *trnK-UUU*, *trnQ-UUG*, *trnS-GCU*, *trnS-UGA*, *trnG-GCC*, *trnM-CAU*, *trnS-CGA*, *trnT-GGU*, *trnE-UUC*, *trnY-GUA*, *trnD-GUC*, *trnC-GCA*, *trnR-UCU*, *trnS-GGA*, *trnT-UGU*, *trnL-UAA*, *trnF-GAA*, *trnV-UAC*, *trnM-CAU*, *trnW-CCA*, and *trnP-UGG*), one gene encoding RNA splicing protein (*matK*), 27 genes encoding ribosomal structural proteins (*rps19*, *rpl2*, *rpl23*, *rps7*, *rps12*, *rps15*, *rpl32*, *rps15*, *rps7*, *rpl23*, *rpl2*, *rps19*, *rps16*, *rps2*, *rps14*, *rps4*, *rpl23*, *rpl33*, *rps18*, *rpl20*, *rps11*, *rpl36*, *rps8*, *rpl14*, *rpl16*, *rps3 and rpl22*), eight genes are ribosomal structural RNAs (*rrn16*, *rrn23*, *rrn4.5*, *rrn5*, *rrn5*, *rrn4.5*, *rrn23*, and *rrn16*), one is translation-related gene (*infA*) and seven unknown genes (*ycf2*, *trnT-CGU*, *ycf1*, *ycf1*, *trnT-CGU*, *ycf2*, and *clpP1*), are found in the chloroplast genome of *A. pectinatum.* Among these genes, 42 of them are duplicated in the IR regions.

To confirm the phylogenetic position of *A. pectinatum*, we downloaded the complete chloroplast genomes of 14 additional species within the Gramineous family from the NCBI GenBank database. All the chloroplast genome sequences of these plant species were aligned using MAFFT v7 (Katoh et al. 2019) and RAxML (v8.2.10) (Stamatakis 2014), and were used to construct a maximum likelihood tree, with *Pharus latifolius* as the outgroup (Figure 1). Phylogenetic analysis shows that *A. pectinatum* is closely clustered with *Kengyilia melanthera* and *Australopyrum retrofractum.* The plants of the *Agropyron* Gaertn are very important wild relative to wheat (*Triticum aestivum* L.), the complete chloroplast genome of *A. pectinatum* also provides useful perspectives into the evolutionary patterns of the *Triticum* genus and further the entire Gramineous family (Vovk and Yakimenko 1973).

**Figure 1.**
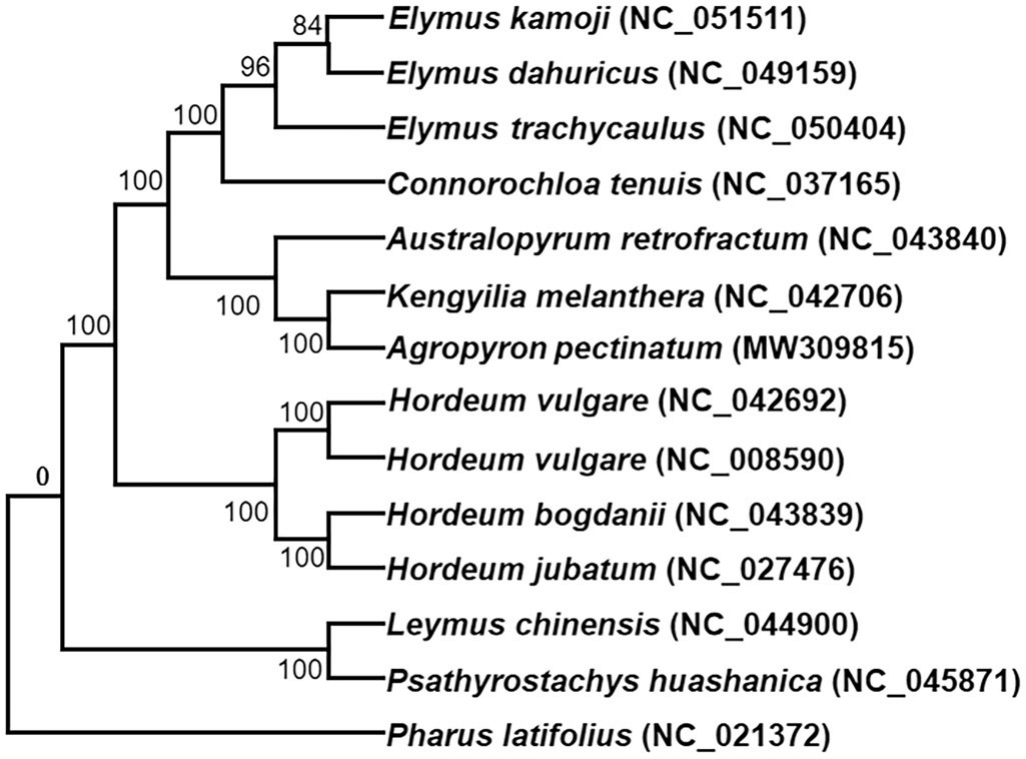
Phylogenetic tree reconstruction using maximum likelihood (ML) method based on the complete chloroplast genome of 14 species. The accession numbers are listed as below: *Australopyrum retrofractum* (NC_043840), *Connorochloa tenuis* (NC_037165), *Elymus dahuricus* (NC_049159), *Elymus kamoji* (NC_051511), *Elymus trachycaulus* (NC_050404), *Hordeum bogdanii* (NC_043839), *Hordeum jubatum* (NC_027476), *Hordeum vulgare* subsp. *spontaneum* (NC_042692), *Hordeum vulgare* subsp. *vulgare* (NC_008590), *Kengyilia melanthera* (NC_042706), *Leymus chinensis* (NC_044900), *Psathyrostachys huashanica* (NC_045871), *Pharus latifolius* (NC_021372), *Agropyron pectinatum* (MW309185).

## Data Availability

The data that support the findings of this study are openly available in NCBI at Genbank under the accession number MW309815 (https://www.ncbi.nlm.nih.gov/nuccore/MW309815). Raw sequencing reads used in this study was deposited in the public repository SRA under the accession number SRR13495513 (https://www.ncbi.nlm.nih.gov/sra/?term=SRR13495513). The associated BioProject and Bio-Sample numbers are PRJNA693585 and SAMN17392497, respectively.
